# Health and social impacts of COPD and the problem of under-diagnosis

**DOI:** 10.1186/2049-6958-9-63

**Published:** 2014-12-06

**Authors:** Stefano Carlone, Bruno Balbi, Michela Bezzi, Marco Brunori, Stefano Calabro, Maria Pia Foschino Barbaro, Claudio Micheletto, Salvatore Privitera, Roberto Torchio, Pietro Schino, Andrea Vianello

**Affiliations:** Pulmonary Department, San Giovanni-Addolorata General Hospital, Rome, Italy; Pulmonary Rehabilitation Department, IRCCS Fondazione Salvatore Maugeri, Veruno (NO), Italy; Endoscopy and Laser Therapy, Respiratory Unit, Hospital of Brescia, Brescia, Italy; Respiratory Pathophysiology and Rehabilitation Unit, Policlinico Umberto I, Rome, Italy; Respiratory Unit, San Bassano Hospital, Bassano del Grappa, Vicenza Italy; Department of Medical and Surgical Sciences, University of Foggia, Foggia, Italy; Respiratory Unit, Mater Salutis Hospital, Legnago, Verona Italy; Centre for Prevention and Monitoring Respiratory Failure, ASP, Catania, Italy; Respiratory Function and Sleep Laboratory, AOU S. Luigi, Orbassano (TO), Italy; Physiopatology Respiratory Unit, General Hospital F. Miulli, Acquaviva delle Fonti (BA), Italy; Respiratory Pathophysiology Division, University-City Hospital of Padova, Padova, Italy

**Keywords:** COPD, Early diagnosis, Indicators, Phenotype, Spirometry, Underestimation

## Abstract

This article deals with the prevalence and the possible reasons of COPD underestimation in the population and gives suggestions on how to overcome the obstacles and make the correct diagnosis in order to provide the patients with the appropriate therapy. COPD is diagnosed in later or very advanced stages. In Italy the rate of COPD under-diagnosis ranges between 25 and 50% and, as a consequence, the patient does not consult his doctor until the symptoms have worsened, mainly due to exacerbations. A missed diagnosis influences the timing of therapeutic intervention, thus contributing to the evolution into more severe stages of the illness. An incisive intervention to limit under-diagnosis cannot act only in remittance (passive diagnosis), but must be the promoter for a series of preventive actions: primary, secondary and rehabilitative. To reduce under-diagnosis, some actions need to be taken, such as screening programs for smokers subjects, use of questionnaires aimed to qualify and monitor the disease severity, spirometry, early diagnosis. There is a consensus regarding diagnoses based on screening of at-risk subjects and symptoms, rather than screening of the general population. In practice, all individuals over 40 years of age with risk factors should make a spirometry test. Screening actions on a national scale can be the following: compilation of questionnaires in waiting rooms of doctor’s offices or performing simple maneuvers to evaluate the expiratory force at pharmacies. It is now widely recognized that COPD is a complex syndrome with several pulmonary and extrapulmonary components; as a result, the airway obstruction as assessed by FEV_1_ by itself does not adequately describe the complexity of the disease and FEV_1_ cannot be used alone for the optimal diagnosis, assessment, and management of the disease. The identification and subsequent grouping of key elements of the COPD syndrome into clinically meaningful and useful subgroups (phenotypes) can guide therapy more effectively. In conclusion, we firmly believe that an early and correct diagnosis can influence positively the progress of the disease (lowering the lung function impairment), decrease the risk of exacerbations, relieve symptoms and increase the patients’ quality of life leading also to a decrease in costs associated to the exacerbations and hospitalization of the patient.

Chronic respiratory diseases are a group of pathological conditions which are currently not sufficiently prevented, underdiagnosed and undertreated [1]. Among these, Chronic Obstructive Pulmonary Disease (COPD) represents a real problem for public health [2]. At European level the prevalence of COPD is approximately 4-7%, with the males and the elderly most affected by this disease condition [3].

## Review

According to a recent study in Italy, COPD affects approximately 3% of the general population, but this prevalence increases with age and in males, such as it reaches 20% in males over 60 years old [4].

The real prevalence of this disease within a population can vary based on the instrument used to identify it, that is according to whether respiratory symptoms, medical diagnosis, or pulmonary function are utilized [5, 6].

The health, social and economic impact of COPD - the third leading cause of death in the United States - is high. In 2010, the global cost associated with COPD in the USA was 50 billion dollars, of which 20bn of indirect costs and 30bn of direct costs [7]. These costs tend to increase in a way directly proportional to the severity of the illness and to moderate and severe exacerbations, especially those requiring hospitalization. Each patient affected by COPD globally cost in 2010 $1,681 if in stage I GOLD, $5,037 if in stage II, and $10,812 if in stage III. The percentage of costs associated with hospitalization increases with the worsening of stages.

In Italy, contrary to the US data, the average annual cost for each patient affected by COPD is 2,724€, out of which 92% is represented by direct costs: in particular, 19.6% is represented by pharmacological therapy, 59.1% by hospitalizations and 6% by instrumental examinations [8].

Exacerbations are a significant part of the natural progression of COPD and are responsible for large portion of deaths, further decline in respiratory function, worsening of the quality of life, and increase in hospitalizations. The ECLIPSE study revealed that the COPD frequent exacerbator phenotype (n. of moderate/severe exacerbations/year ≥ 2) represents approximately 30% of all COPD patients [9].

Hospitalizations are generally the consequence of severe exacerbations of COPD, and have a greater impact on health costs [10].

One of the latest pharmacoeconomic studies regarding this aspect points out that for each patient that has two or more exacerbations/year [11], reducing exacerbations with an appropriate treatment means reducing hospitalizations and thus related costs.

## The problem of under-diagnosis

Currently, COPD is diagnosed in later or very advanced stages. In Italy the under-diagnosis of COPD oscillates between 25 and 50% and the results of some epidemiological surveys conducted in the Padania Delta and in Pisa-Cascina (zones of Italy) confirm the international observations in this respect [12].

In Spain, it has been observed that only 60% of patients with chronic respiratory symptoms go to a doctor and only 45% of them are then directed to a spirometry test [13].

Respiratory symptoms are often not felt or are underestimated by the patient, who considers his symptoms as physiological consequences of age or smoking habits [14, 15]. As a consequence, the patient does not consult his doctor until his symptoms are aggravated, mainly due to exacerbations. Also the general practitioner (GP) can underestimate the situation in these occurrences, diagnosing the episode only as an acute event rather than an epiphenomenon of an unrecognized chronic problem, thus neglecting to look further into the clinical history. The result is that up to 80% of subjects affected with airways obstruction, according to the GOLD spirometric criteria, has never had a diagnosis of COPD, and even among those with severe obstruction fewer than half have already been diagnosed [16]. A missed diagnosis influences the timing of therapeutic intervention, thus contributing to the evolution into more severe stages of the illness.

A late diagnosis may recognize many causes, of which the most important are:

1. poor implementation of screening programs in young smokers (40 to 55 years old)
2. little use of the GOLD or other questionnaires on a general medicine level
3. not enough use of the spirometry both at general medicine and pulmonologist level
4. low perception of symptoms in the initial phases of the sickness.

Given the above mentioned epidemiological data, an incisive intervention to limit under-diagnosis cannot act only in remittance (passive diagnosis), but must act as the promoter for a series of preventive actions: primary, secondary and rehabilitative. For this reason, the “Schema di piano sanitario nazionale 2011-2013” (National Health Plan 2011-2013) [17] establishes that the approach to managing chronic respiratory illnesses, able to reconcile optimal health assistance with sustainable public spending, is represented by “primary prevention and diagnosis as early as possible, with standardized instruments, followed by appropriate and timely therapies, able to prevent or delay invalidity, treating those with chronic illnesses in their own territory as much as possible*”.* The document, therefore, aims at the precocity of diagnosis rather than at the promotion of informative and educational interventions against the main causes, such as smoking, or activation of programs to reduce environmental and professional risks.

To reduce under-diagnosis, some actions need to be taken:

1) *Screening programs for at-risk subjects: smokers*

In a setting of Swedish general practitioners, 27% of smokers aged between 40 and 55 years, showed bronchial obstruction (85% mild form, 13% moderate form and 2% severe form) [18]. Similar results were seen in Spain [19] and Holland [20]. More recently, a screening program in Germany, conducted on smokers aged between 40 and 60 years, identified 19% with COPD in GOLD stage I [21].

It has been shown that a minimal anti-smoking educational approach reduces the percentage of smokers from 24% to 16% [22].

2) *Use of the GOLD questionnaire*

The use of the GOLD questionnaire appears to be very helpful at the general medicine level.

According to the International Primary Care Respiratory Group, the systematic use of the GOLD questionnaire at the general medicine level allows for early identification of the stages of the illness [23].

3) *Spirometry*

The use of spirometry in smokers, or those exposed to atmospheric and/or environmental pollutants, who show productive cough and dyspnea, allows for early identification of the illness.

### Early diagnosis

Early diagnosis is important as it allows for immediate action against the causes of the illness (mostly smoking) so that the progression of the respiratory pathology towards more severe and invalidating levels can be delayed or stopped [20, 24].

Smoking cessation and long-term oxygen therapy in the advanced phases of the illness represent the only therapeutic measures proved effective in modifying the natural progression of COPD and improving the survival rates [25]. Furthermore, pharmacological therapy and rehabilitation reduce the risk of exacerbations and improve the quality of life [24]. An early start to treatment reduces the effects of the illness on quality of life and the risk of depression following diagnosis. As these psychological factors influence patients’ adherence to therapy as well as their quality of life, the doctor should know how to recognize and diagnose them [26].

Early diagnosis is responsibility of GP, who should understand the dimensions of the problems caused by respiratory diseases and in particular by COPD. For example, a common practice should be to record the smoking habit of each patient in the clinical file as well as the presence/absence of cough, catarrh, or dyspnea. For each patient who is a heavy smoker (pack/year > 15 is considered to be at risk) or who has such symptoms, a spirometry should be facilitated and strongly advised.

In this regard, a document jointly published by the Italian Respiratory Societies together with a GP’s Society and endorsed by the Italian Health Ministry [27], recommends that all at-risk subjects undergo a simple spirometry, while those with respiratory symptoms be examined with global spirometry.

### From clinical suspicion to diagnosis: screening of at-risk subjects

There is a consensus regarding diagnoses based on “screening of at-risk subjects*”*, rather than screening of the general population. The latter would not be cost effective, due to the fact that the prevalence of COPD cases remains modest among the general population, while it is decisively higher in the group of smokers over 40 years of age.

This approach is advised by the World Health Organization (WHO) in its document on the Global Alliance for Respiratory Disorders (GARD), which suggests a spirometry for each at-risk individual [28].

In practice, this means that all individuals over 40 years of age with risk factors should undergo a spirometry test. Besides smoking, by far the most important cause of COPD, professional exposure to dust particles and other inhalable noxious substances for the respiratory apparatus should be considered, as well as atmospheric pollution, and not only for those who live in big cities.

What are then the factors that hinder the screening in at-risk subjects?

The current waiting lists for respiratory function tests, which in many regions are no longer than 5-6 weeks, do not seem to represent the critical factor for the delay in diagnosis or under-diagnosis of COPD, nor does the distance of the patients from specialized centers. COPD develops over many years and decades and, therefore, there is not an “urgency” for an evaluation but rather a cogency for the evaluation to be done. In other words, it is not important for the spirometry to be done from one day to the next, but it is important that it is done. In this regard, the relative brevity of the waiting list for the spirometry is an indirect index of under-diagnosis of a pathology with a high prevalence. In medical groups, one could foresee the execution of spirometry in doctor’s offices with qualified hospital personnel to control the quality of the tests, and even utilizing sponsorships from associations and private individuals.

On the other hand, there seems to be the need for a better awareness of the problems concerning chronic respiratory diseases, and in particular COPD, and how they will worsen in our society in the medium term. The “COPD problem” needs to be put on the agenda for healthcare planning at a central, regional and local level, with integrated and targeted actions.

In this scenario, where early diagnosis of COPD is a shared objective at all levels, we can hypothesize various approaches.

Screening actions on a national scale can be translated into the compilation of questionnaires in waiting rooms of doctor’s offices or in pharmacies, in order to find risk factors such as smoking habits, coughs, sputum or dyspnea [29]. Pharmacies should also play a facilitating role in early diagnosis: similarly to the measurements of glycemia, blood pressure and other blood chemistry, all easily available, a measurement of FEV_1_ could be done simply and quickly, with a subsequent notification to the GP.

If there was more awareness of the “COPD problem”, a preventive campaign aimed at heavy smokers, with an invitation to undergo a spirometry and clinical evaluation every three-five years could be foreseen, much like that which is already done in the oncological sector and is commonly accepted by the population for the screening of certain types of tumors.

In general, mass communication instruments (for example commercials) could launch a campaign to sensitize the public regarding respiratory problems.

### After the COPD diagnosis

The first phases of the disease and the less complicated cases should be the prerogative of the general practitioner, who, therefore, becomes the pin around which the initial diagnosis and therapies rotate. The paucisymptomatic patient, with little obstruction at spirometry, with rare or no exacerbations, especially if motivated and aware, should be managed at the primary care level. Smoking cessation, prevention of exacerbations with vaccinations, adequate lifestyle (for example maintaining or starting regular physical activity), and pharmacological therapy with bronchodilators, where needed or continuously, are key to this management. In less severe cases (stage I or stage A GOLD) the follow up could be every year, with a repetition of the spirometry associated with a clinical re-evaluation.

The patients with more advanced forms of the illness, both from a functional and symptomatic standpoint, those troubled with comorbidity (ex. cardiovascular), or those patients with respiratory insufficiency or frequent exacerbations, should be referred to the pulmonologist.

It seems, therefore, evident that what above described represents a management model with several fundamental characteristics: a sharing of the progression of the patient, a close contact between GP and pulmonologist, an educational approach and, where possible, a setting of self-management by the patient, along with a well-thought out, gradual and therefore cost efficient use of healthcare resources.

### COPD: a complex of multiple disorders

The call for the development of a new taxonomy for the disorders of airflow obstruction has originated from the recognition that asthma and COPD are not a single disease, but rather syndromes including multiple separate disorders. A better understanding of the distinct disorders of airways disease has the potential to inform on underlying mechanisms, risk factors natural history, monitoring and most importantly, treatment. The history of the guidelines of treatment of COPD is an example of the simplification of a complex disease. The Venn diagram included in the 1995 American Thoracic Society statement for the management of COPD [30] reflected the complexity of the disease and its different clinical presentation. The limited alternatives for pharmacological treatment at that time made it unnecessary to identify the different types of patients for clinical practice. The evolution of the concept of one-treatment-fits-all led to the selection of pharmacological treatment based almost exclusively on the severity of airflow obstruction introduced in the first GOLD documents.

The diagnosis, assessment and management of COPD are currently facing an important dilemma. On the one hand, COPD is defined by the presence of airflow limitation that is not fully reversible, and its treatment is mostly guided by the severity of this limitation. On the other hand, it is now widely recognized that COPD is a complex syndrome with numerous several pulmonary and extrapulmonary components. Importantly, significant heterogeneity exists with respect to clinical presentation, physiology, imaging, response to therapy, decline in lung function, and survival. As a result, there is consensus that airways obstruction as assessed by FEV_1_ by itself does not adequately describe the complexity of the disease and that FEV_1_ cannot be used alone for the optimal diagnosis, assessment, and management of the disease [31].

The recent revision of the GOLD initiative has moved forward and changed the paradigm proposing a treatment directed by the intensity of symptoms, measured by the modified medical research council dyspnea scale and/or the COPD assessment Test, and the risk of poor outcomes, identified by the degree of airflow obstruction and/or the frequency of exacerbations [32].

### What do we mean by “COPD phenotype”?

The identification and subsequent grouping of key elements of the COPD syndrome into clinically meaningful and useful subgroups (phenotypes) that can guide therapy more effectively is a potential solution of the dilemma.

A group of expert recently proposed the following variation on the traditional definition of a phenotype: “a single or a combination of disease attributes that describe differences between individuals with COPD as they relate to clinically meaningful outcomes (symptoms, exacerbations, response to therapy, rate of disease progression, or death)” [33].

In other words, it is proposed that phenotypes in COPD should have real predictive value. The phenotype should be able to classify patients into subgroups with prognostic value and to determine the most appropriate therapy to achieve better results from a clinical standpoint. Many studies have attempted to identify and quantify the prevalence of different phenotypes of COPD using populations of various source and severity. There is still no consensus on the number and definition of different COPD phenotypes. There must be a compromise between the oversimplification of the term COPD as a definition that encompasses the entire spectrum of patients with incompletely reversible airflow obstruction largely caused by smoking, and the complexity of considering each patient individually as an orphan disease [34].

### Phenotypes of clinical interest in COPD

According to the responses to pharmacological treatment, the recent Spanish guidelines for the treatment of COPD [35] have proposed four different phenotypes characterized by the classical types of emphysema, chronic bronchitis, exacerbators and patients with overlap COPD-asthma. The proposed phenotypes in Guia Espanola de la EPOC are as follows: 1) infrequent exacerbator with either chronic bronchitis or emphysema; 2) overlap COPD-asthma; 3) frequent exacerbator with predominant emphysema; 3) frequent exacerbator with predominant chronic bronchitis.

The overlap COPD-asthma phenotype is associated with enhanced response to inhaled corticosteroids due to the predominance of eosinophilic bronchial inflammation [36]. The Spanish guidelines have also recognised this phenotype and a consensus document has been generated with diagnostic criteria to identify patients with overlap COPD asthma [37]. Among these criteria, the most important are the history of previous asthma before the age of 40 years, the demonstration of eosinophilic inflammation in sputum or increased peripheral eosinophilia, and enhanced reversibility in airflow obstruction after the bronchodilator test. Only 20% of overall patients with respiratory obstructive diseases (i.e. COPD plus asthma) over 64 years old belong to this category where a combined therapy may be beneficial [38].

Looking closer to the prevalence of these different phenotypes, it crystallizes that the majority of the patients belong to the group “infrequent exacerbator with either chronic bronchitis or emphysema”. In this regard, the observational ECLIPSE study, lasted 3 years, with over 2,000 patients enrolled, provided substantial data to demonstrate that the vast majority (70%) of the patients with moderate-to-very severe COPD had less than 2 exacerbations per year [9]. Of note, this study excluded mild COPD patients: therefore, it is possible to assume that among mild patients the percentage of infrequent exacerbators is even higher. The cornerstone of the therapy for these patients are long-acting bronchodilators as also recommended by international guidelines. According to the same study, 30% of the moderate-to-very severe patients are frequent exacerbators where, in addition to the bronchodilation therapy, inhaled corticosteroids are recommended.

### Evaluation of comorbidities

The cardiovascular comorbidities, such as ischemic cardiac disease, systemic hypertension, hearth failure, stroke, pulmonary hypertension are the most important in terms of prevalence, prognosis and mortality and have in common with COPD the risk factors and the age. Ageing of the population, in fact, increases the prevalence of chronic diseases, including cardiovascular and pulmonary diseases, cancer and metabolic syndrome. Almost half of all elderly people (> 65 yrs) have at least three chronic medical conditions and one fifth have five or more [39]. But the most relevant consideration about the comorbidities is that they affect health outcomes in COPD. Patients with COPD mainly die of non respiratory diseases such as cardiovascular diseases (25-27%), cancer (mainly lung cancer 20-33%) and other causes (30%) [40, 41]. Respiratory failure due to COPD exacerbations accounts only for 4-35% of deaths.

## Conclusions

This article deals with the prevalence and the reasons of the underestimation of COPD in the population. Furthermore, some suggestions were given on how to overcome the obstacles to make the correct diagnosis in order to provide the patients with the appropriate therapy. We firmly believe that an early and correct diagnosis can positively influence the progress of the disease (lowering the lung function impairment), decrease the risk of exacerbations, relieve symptoms and increase the quality of life of the patients leading also to a decrease in the costs related to the exacerbations and hospitalization of the patient.
